# Physicochemical properties of otic products for Canine Otitis Externa: comparative analysis of marketed products

**DOI:** 10.1186/s12917-023-03596-2

**Published:** 2023-02-09

**Authors:** Yunmei Song, Sadikalmahdi Abdella, Franklin Afinjuomo, Emily Josephine Weir, Jin Quan Eugene Tan, Peter Hill, Stephen W. Page, Sanjay Garg

**Affiliations:** 1 Centre for Pharmaceutical Innovation(CPI), Clinical and Health Sciences, University of South Australia, Adelaide, SA 5000 Australia; 2 Small Animal Specialist Hospital, Adelaide, SA 5067 Australia; 3 Neoculi Pty. Ltd., Burwood, VIC 3215 Australia

**Keywords:** Canine otitis externa, Formulation development, Physicochemical properties

## Abstract

**Background:**

Otitis externa is a commonly diagnosed dermatological disorder in canines. The pathogens primarily involved in canine otitis externa (COE) include *Staphylococcus pseudintermedius, Pseudomonas aeruginosa, Proteus mirabilis,* and *Malassezia pachydermatis*. As COE tends to be superficial, medications delivered topically are often effective and practical in managing the condition. As such, there is a wide variety of approved topical products currently available in the market. The efficacy of topical dosage forms can be dependent on various factors such as the pharmacology of active constituents and the physicochemical properties of the formulation, including pH, viscosity, spreadability, and bio-adhesion. Currently, there is a lack of published literature available on the optimal properties of topical COE products. In this study, we compared the physicochemical properties of nine commercially available otic veterinarian products in Australia used clinically to manage COE.

**Results:**

Based on our comparative analysis, the pH (6.26 ± 0.04) of an aqueous-based product was similar to a healthy dog’s external auditory canal. Products containing polymers exhibited higher viscosity and bio-adhesion. Spreadability was inversely related to viscosity and Osurnia ® a product with high viscosity demonstrated the lowest spreadability. Aqueous-based otic products showed better syringebility whereas oil-based systems required higher force to expel the products. Variability in droplet size was noted. Derm Otic, Baytril Otic, and Aurizon Ear Drops had the lower standard deviation which indicates they would give a more consistent dose.

**Conclusions:**

Findings from this work provide considerations for industry researchers or formulation scientists working in the area of otic dosage formulations.

**Supplementary Information:**

The online version contains supplementary material available at 10.1186/s12917-023-03596-2.

## Background

Canine Otitis Externa (COE) refers to an inflammatory condition of the external auditory canal [1]. It is a commonly diagnosed dermatological disorder in dogs with an incidence of 5–20% and a prevalence of 8.7%-20% [1–5]which varies with the breed of dog [6, 7]. COE is classified as acute and chronic depending on the duration of the inflammatory condition. Chronic COE lasts for 3 months or longer and is usually associated with the presence of an unresolved underlying cause such as skin allergies, or the development of perpetuating factors such as hyperplasia of the EAC, resistant bacteria, or progression to otitis media. Additionally, treatment failure of acute COE can occur, as the majority of COE cases become either recurrent or chronic [2–4, 8]

The aetiology of COE is multifactorial and associated with primary, secondary, predisposing, and perpetuating factors. Primary factors such as keratinization, skin allergies, immune-mediated disorders, and foreign bodies initiate infection by affecting the skin or the ear canal itself whereas secondary, predisposing, and perpetuating factors contribute to the progression of the otitis externa [9, 10]. Common pathogens in COE include *Pseudomonas aeruginosa, Staphylococcus pseudintermedius, Malassezia pachydermatis,* and *Proteus mirabilis* [11]. Biofilm-producing bacteria such as *Staphylococcus* and *Pseudomonas* often lead to persistent infection as eradication requires disruption of the biofilm. Complicating the aetiology, *Malassezia* often causes an allergic response, leading to significant discomfort and pruritus [12].

Topical therapies are the mainstay for the management of COE. Commonly found active constituents in COE products include glucocorticoids, antibacterial agents, and antifungal agents. Manual cleaning is also recommended to remove debris prior to the application of the topical product to ensure contact with the affected site in the canine ear cavity. Nevertheless, recurrent or chronic otitis is challenging to treat and successful management requires addressing all the underlying factors responsible for the recurrence of otitis [13].

Apart from the pharmacology of active constituents in COE preparations, the physicochemical properties of the formulation can influence the delivery of the drugs to the target site. Formulation properties such as adhesion and spreadability could affect the effectiveness of topical formulations. Poor bio-adhesion results in reduced retention times at the affected site and increased risk of product lost upon head shaking and scratching by the canine following administration. Good spreadability properties are also integral in COE products for ease of administration and appropriate coverage of the infected area [2]. There is an inverse relationship between spreadability and viscosity [14], whilst residence time and product retention can be improved by increasing viscosity [15]. This presents a conundrum when producing COE preparations due to the previously mentioned interactions between residence time and spreadability with product viscosity.

Recently, there has been considerable research on the use of thermosensitive in situ gels to improve otic delivery systems. In situ -gel-based dosage forms begin as free-flowing liquid solutions which undergo a rapid phase transition into a gel when applied at the site of infection. The phase transition occurs due to a change in pH and/or temperature [16]. In situ gels have demonstrated promising improvements and overcome common drawbacks of otic drug delivery including short residence time due to leakage of medication from the ear, inconvenient administration, and poor compliance [15]. Foam technology is another method to improve the residence time of the drug in the ear. Foams are a light frothy mass of bubbles that are either formed from the liquid or on the surface of a liquid [17]. The formation of the foam not only reduces the fluidity of the liquid but can result in a higher area of coverage compared to a solution. Marom et al. compared the safety and efficacy of foam and solution-based ciprofloxacin for the treatment of otitis externa [17] in humans. Although no significant difference was observed in efficacy among the two formulations, foam-based products showed superior results with ease of application and physical appearance which might influence long-term treatment outcomes [17].

Whilst there are several topical formulations commercially available for the treatment of COE, there are no “gold standard criteria” providing guidance for formulation development. For the first time in this study, we have evaluated important physicochemical properties of selected marketed otic products used for the treatment of COE (Table 1) in Australia. The outcomes of this study will provide considerations for formulation scientists to impute when designing a topical drug formulation for COE products.

**Table 1:** List of the Selected Commercial Otic Products with their Active Ingredients

Commercial COE Products	Manufacturers	Active Ingredients		
		Antifungal	Antibacterial	Corticosteroid
**Surolan**	Elanco	Miconazole Nitrate	Polymyxin B Sulphate	Prednisolone Acetate
**Derm Otic**	Troy Laboratories	Miconazole Nitrate	Polymyxin B Sulphate	Prednisolone Acetate
**Mometamax**	MSD animal health	Clotrimazole	Gentamicin Sulfate	Mometasone Furoate Monohydrate
**Easotic**	Virbac	Miconazole Nitrate	Gentamicin Sulphate	Hydrocortisone Aceponate
**Canaural**	Dechra	Nystatin	diethanolamine fusidate, Framycetin Sulphate	Prednisolone
**Osurnia**	Elanco	Terbinafine	Florfenicol	Betamethasone Acetate
**Baytril Otic**	Bayer		Enrofloxacin, Silver Sulfadiazine	
**Aurizon Ear Drops**	Vetoquinol	Clotrimazole	Marbofloxacin	Dexamethasone Acetate
**Apex PMP**	Apex/ Dechra	Miconazole Nitrate	Polymyxin B Sulphate	Prednisolone Acetate

## Method

### pH determination

The pH of the sole aqueous otic product (Baytril Otic) was measured using a pH meter (Thermo Scientific *Orion PerpHecT ROSS*™ Combination *pH Micro Electrode*). The reading was performed in triplicates. The results were reported as mean and standard deviation.

### Determination of viscosity

Rheosys Merlin Viscometer/Rheometer (VR) (Scientex Pty Ltd, Melbourne, Victoria, Australia) viscometer was used to determine the viscosity of the otic products. The viscosity was evaluated at 25 ℃ (room temperature) and 38.9 ℃* (*the temperature of the EAC in cases of COE). The temperature of the canine EAC is usually between 38.2 to 38.4 ℃ but during infection, the EAC temperature increases to 38.9 ℃ [18]. One of the products, Osurnia, is required to be stored in the refrigerator, therefore, it was tested at three different temperatures 4℃, 25℃, and 38.9℃*.* The instrument parameters used for the viscosity measurement are given in Table 2.

**Table 2:** Parameters & Setting of aViscosity Tester for Evaluation the Selected Commercial COE Products

Parameter	Setting
Measuring System	15 mm Parallel Plate 0.5 mm Gap
Temperature-controlled parallel plate	25 °C with 15 s of thermal equilibrium
Direction	Up
Steps	10
Log/Lin	Linear
Delay	60 s
Integration	5 s
Pre-test speed	10 (1/s) for 20 s

### Determination of bioadhesive properties

**Fig. 1:**
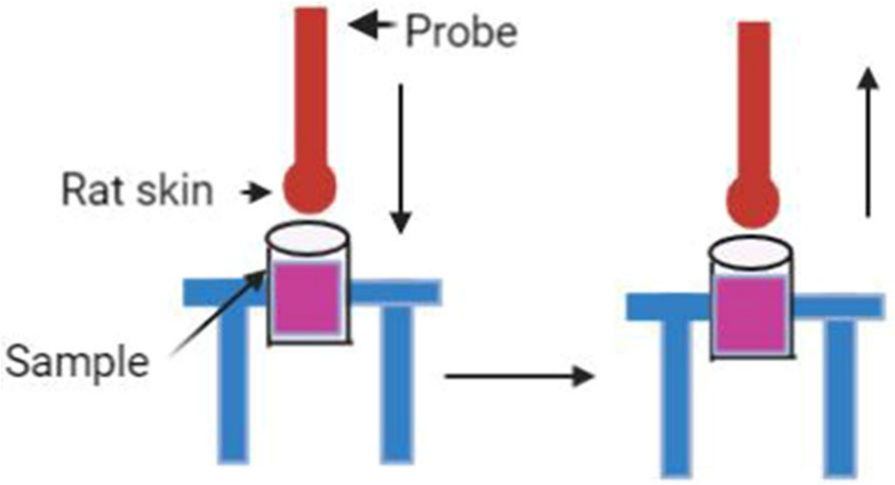
A schematic diagram of the texture analyser used to evaluate bioadhesion

Bioadhesion provides a surrogate measure for the residence time of the otic products at the site of application and ultimately determines the extent of drug absorption. Bioadhesion was evaluated using the TA.XT Plus Texture Analyser (Stable Micro Systems). The texture analyser setting used to test the stickiness of adhesive gum was used in this study (Table 3). Rat skin (subject to collection approval by way of institutional scavenged tissue notification) was used as a bioadhesive surface. It was attached to a cylindrical probe with a rubber band and 0.2 g of the sample product was placed in the center of a weigh boat which was fixed to the base of the texture analyser with adhesive putty (Fig. 1). The probe, with skin attached, was lowered at a constant speed of 0.5 mm/sec into the sample product. The contact time was 3 s and a constant downward force of 500 g before rising to the return distance of 10 mm at a constant speed of 1.0 mm/sec. The detachment force recorded by the *Exponent* software was used to determine and compare the differences in bioadhesive properties of commercial COE products. For each product tested, the rat skin was replaced with a fresh piece. The test was performed in triplicate. Notification of the use of scavenged tissues was made as per the protocol to the animal ethics committee of the University of South Australia before commencing the experiment.

**Table 3:** Texture Analyser Settings for Evaluation of Bioadhesion of the Selected Commercial COE Products

Parameters	**Setting**
Pre Test Speed	0.5 mm/sec
Test Speed	1.0 mm/sec
Post Test Speed	1.0 mm/sec
Applied Force	500 g
Return Distance	10 mm
Contact Time	3.0 s
Trigger Type	Auto
Trigger Force	5.0 g

### Spreadability evaluation

Spreadability was evaluated as described by Dantas et al. [19]. Briefly, a large glass slide was placed onto 5 mm lined graph paper where the center of the slide was marked. The glass slide with the graph paper underneath was placed onto a weighing scale and the scales zeroed. 100 mg of the product was placed in the center of the slides. A second glass slide previously weighed at 240 g, was then placed gently directly on top of the first glass slide containing the product. The slides were left for 30 s and then the diameter was measured from left to right using an mm-marked ruler. The spreadability of each product was tested three times.

### Syringeability determination

Syringability of the products was tested using the TA.XT Plus Texture Analyser (Stable Micro Systems) using a 1 mL syringe without a needle (Terumo syringe) at room temperature (22 ± 2 °C). Tests were conducted as quadruplicates for each product, and the testing sample was drawn up to 0.5 mL in the syringe. The test mode of the apparatus was set to compression; the distance and test speed of compressions were 25 mm, and 5.00 mm/sec respectively.

### Droplet size determination

**Fig. 2:**
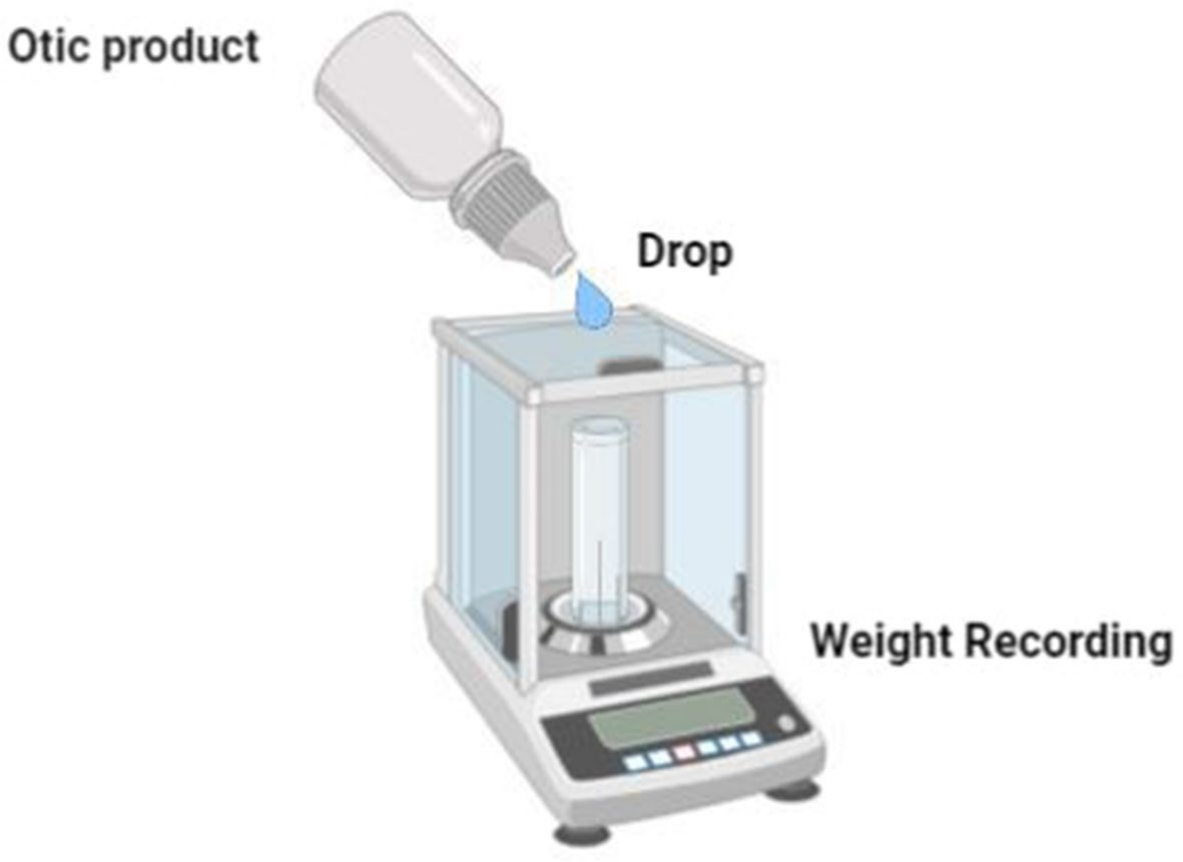
A schematic diagram of droplet size determination

A weighing scale was used to evaluate the droplet size. Ten drops obtained directly from each otic product as packed were individually measured for each product. The average droplet weight and standard deviation were calculated. The droplet mass is assumed to be proportional to droplet size (Fig. 2).

## Result and discussion

### pH value

The normal canine skin pH ranges from 5.5–7.2, slightly more alkaline compared to the pH of human skin which is in the range of 4–6 [20–22]and similar to the pH of the epithelium of the EAC of dogs with normal ears (4.6–7.2) [23]. In the EAC of dogs with chronic COE, the pH tends to have a higher range of (6–7.4), whilst in acute COE the pH tends to be on the lower side ranging from 5.2 to 7.2 [23]. From the range of commercial products we have selected from the study, only Baytril Otic ® is aqueous-based with an approximate pH of 6.26 ± 0.04. This is an ideal property as it is similar to the pH range of a healthy dog’s EAC, hence it is less likely to disrupt the micro-environment required for the maintenance of healthy skin. Furthermore, the EAC is well buffered and the impact of any administered product is likely to be transient [24].

### Viscosity evaluation

Viscosity is a vital property of pharmaceutical preparations and it is commonly modified by formulation scientists to control the retention times of topical drugs at the site of application. The viscosity of the evaluated products varied significantly. Most of the products had a viscosity in the range of 0 – 1 Pa.S. Osurnia ® exhibited the highest viscosity at 4 °C ( 15.5 Pa.S), 5 times greater than Surolan ® (2.8 Pa.S). The viscosity of Osurnia ® was temperature-dependent and was significantly reduced at 39 °C, this can result in reduced retention time in an infected EAC. The high viscosity of Osurnia ® could be related to the polymer, hydroxypropyl methylcellulose (HPMC) present in the formulation [25]. Baytril Otic ® and Dermotic ® demonstrated the lowest viscosity values at both temperatures. Compared to the other products in the study, shear rates and RPM were larger, while torque was smaller, indicating a low amount of shear resistance and therefore greater flow. Both of these products are formulated as thin liquid emulsions. Baytril Otic is a water-based emulsion whilst Dermotic is an oil-based emulsion (Table 8).

The viscosity of formulations can be enhanced by increasing the concentrations of polymers within the formulation and using excipients with higher viscosity [26]. Higher viscosity promotes retention of product at the site of application, however, this can lead to poor spreadability [14].

### Bioadhesion evaluation

Bioadhesion studies were conducted to study the retention time of the products at the site of application. Among the evaluated commercial products (Table 4), the product Osurnia ® had the highest detachment force (124.3 g), a marker of bioadhesive strength. The bioadhesive strength of Osurnia ® could be attributed to its gel formulation and the high viscosity of the polymer used, HPMC. HPMC is known to have strong bioadhesive property which is not influenced by pH. The hydrophilic nature of HPMC contributes to higher swelling of the polymer and therefore a higher extent of bioadhesion [27]. In contrast, Baytril Otic ® and Aurizon ® ear drops exhibited low detachment force (14.6 g and 17.4 g, respectively) indicating poor bioadhesive properties. This is likely due to the formulation of the products as both Baytril Otic ® and Aurizon ® ear drops are not gel-based and do not incorporate polymers within their formulation.

**Table 4:** Detachment force values of the Selected Commercial COE Products (mean ± SD, *n* = 3*)*

Product No	Product Name	Detachment Force (g)
**1**	Surolan	38.0 ± 7.6
**2**	Dermotic	69.4 ± 8.1
**3**	Mometamax	77.9 ± 7.9
**4**	Easotic	33.1 ± 3.0
**5**	Canaural	29.7 ± 7.2
**6**	Osurnia	124.3 ± 25.5
**7**	Baytril Otic	14.6 ± 2.4
**8**	Aurizon Ear Drops	17.4 ± 5.1
**9**	Apex PMP	37.2 ± 2.7

Formulations with good bioadhesive properties will demonstrate better retention time on the infected area of the EAC, allowing the medication to be delivered efficiently which directly improves the efficacy of the product [28]. Several factors investigated in this study so far seem to influence the bioadhesiveness of otic formulations such as the type of polymer used, pH, and temperature.

### Spreadability evaluation

A topical formulation with good spreadability properties facilitates even application of the product to the skin optimising drug delivery [19]. The spreadability of the otic drops shows an inverse relationship to viscosity. These phenomena are confirmed in the findings of our study, for example, Osurnia ® a product with high viscosity demonstrated the lowest spreadability with an average value of 35 mm. While products with poor spreadability have good retention properties, poor spreadability can affect the ease of application. Hence the viscosity of the product has to be finely balanced to accommodate for appropriate spreadability and product retention (Table 5).

**Table 5:** Spreadability of the Selected Commercial COE Products (Mean ± SD, *n* = 3)

Product Number	Product Name	Spreadability	
		**Amount used for testing (mg)**	**Diameter of spread area (mm)**
**1**	Surolan	109 ± 1.2	54 ± 0.6
**2**	Derm Otic	105 ± 3.6	66 ± 2.5
**3**	Mometamax	105 ± 3.6	56 ± 4.5
**4**	Easotic	109 ± 11.0	46 ± 1.5
**5**	Canaural	112 ± 4.2	79 ± 1.5
**6**	Osurnia	100 ± 6.2	35 ± 1.2
**7**	Baytril Otic	107 ± 4.7	69 ± 7.2
**8**	Aurizon Ear Drops	106 ± 5.7	50 ± 2.6
**9**	Apex PMP	108 ± 7.6	52 ± 1.0

### Syringeability evaluation

Studies have reported the difficulty of achieving consistent dosing using the proprietary containers, syringe administration has been suggested to facilitate the delivery of an appropriate amount of some products [29]. The syringeability of a topical product is an important factor that requires consideration during formulation development. Syringeability measures the force required to expel the product from a syringe [30]. The syringeability force can serve as a surrogate for the force required to administer the product from its container. The lower the force required to expel the product, the better the syringeability. Results from syringeability studies are largely determined by the rheological behaviour of each product. A product exhibiting shear thinning behaviour will present greater syringeability than other flow behaviours [31].

Baytril Otic ® had the lowest syringeability force (137 g) followed by Osurnia ® (141 g). The small syringeability force required for these products suggests they have good flow properties enabling easier administration of the otic product from the proprietary container (Table 6).

**Table 6:** Syringeability Force Required from the Selected Commercial COE Products (Mean ± SD, *n* = 4)

Product No	Product name	Syringeability Force (g)
**1**	Surolan	293 ± 43
**2**	Derm Otic^a^	1546 ± 155
**3**	Mometamax	970 ± 469
**4**	Easotic	1400 ± 254
**5**	Canaural	377 ± 165
**6**	Osurnia	141 ± 22
**7**	Baytril Otic^a^	137 ± 7
**8**	Aurizon Ear Drops	1199 ± 51
**9**	Apex BMP	1028 ± 248

^a^ Three replicates were used as adequate sample wasn’t obtained

Within our study Baytril Otic ® is the only product formulated as a water-based emulsion and it was found to be the least viscous product. Our findings from the viscosity studies (Supplementary Table 1), found that Baytril Otic ® had a high shear rate and low syringeability force which translates into good flow properties. Conversely, Osurnia ® a gel-based product was found to be highly viscous. Despite its high viscosity Osurnia ® demonstrated comparable syringability to Baytril Otic ®, the product contains HPMC, a polymer that exhibits shear thinning behaviour [32]. Shear stress causes the polymer network of HPMC to align in the flow direction, therefore as the product flows at a high shear rate through the syringe, the viscosity decreases [31]. Derm Otic ® and Easotic had the highest syringeability force (1546 g and 1400 g respectively) indicating poor fluidity and will likely be more difficult to dispense the product from the container with a syringe. From the viscosity studies, Easotic has a slightly larger viscosity than Derm Otic and a low shear rate which reflects the large syringeability force value. Derm Otic is a thick, oily suspension which explains the high syringeability force. Derm Otic shows good bioadhesive properties hence it can be considered a ‘sticky’ product. Although it has low viscosity and a high shear rate, the adhesion of the product to the syringe causes a high syringeability force.

### Droplet size evaluation

Droplet size is an important parameter to consider when administering ear drops as it influences the amount of drug delivered per drop. Ideally, the droplet sizes from the dispensing bottle should be consistent to ensure that an equal dose is delivered each time. A study by De et al. [33] using betamethasone ear drops found significant inter-person variability in the dose administered when dispensed. Table 7 shows the droplet size of the nine otic products investigated.

**Table 7:** Average Droplet Size of the Selected Commercial COE Products (Mean ± SD, *n* = 10)

Product Number	Product Name	Droplet Size (mg/drop)
**1**	Surolan	23 ± 3.8
**2**	Derm Otic	34 ± 1.7
**3**	Mometamax	20 ± 3.4
**4**	Easotic	27 ± 3.9 Use dosage pump for application
**5**	Canaural	25 ± 3.7
**6**	Osurnia	30 ± 6.2 One tube per application
**7**	Baytril Otic	28 ± 1.4
**8**	Aurizon Ear Drops	28 ± 1.6
**9**	Apex PMP	26 ± 4.7

From the result presented in Table 7, the Mometamax droplet size is less than others and Derm Otic offers the largest average droplet size. Derm Otic, Baytril Otic, and Aurizon Ear Drops had the lower standard deviation which indicates they would give a more consistent dose. A larger droplet size suggests that a larger amount of product is administered on each application which might result in unwanted side effects. Furthermore, the variability of each dosing amongst individuals (e.g. different owners, ages, and sexes) can lead to the variant drop application for the required amount of drugs.

Among the commercial COE products tested, Easotic is packaged with a pump device designed to control and ensure that a precise dose is delivered from the pump each time. Each dose of Osurnia is packaged individually in a small tube containing 1 mL of product where one whole tube is to be used per dose. The premise of these dosing systems would indicate a reduction in variability between doses. However, further testing of these systems is required to properly determine whether variation exists and whether it is statistically significant.

### Systemic absorption of drugs

As COE is a superficial infection, drug penetration into the systemic circulation is unwanted as it may result in adverse effects. Most of the evaluated otic products were oil-based formulations (Table 8) and will likely possess low water activity, higher osmotic pressure, and decreased skin penetration. Osmotic pressure has been shown to affect drug penetration. Therefore, it is important to make an appropriate choice of vehicle to minimise systemic absorption of the topical formulations [34]. Mills and colleagues have highlighted the impact of the choice of vehicles and application sites on transdermal drug penetration through canine skin [35]. They investigated the penetrability of testosterone using 3 different vehicles phosphate-buffered saline (PBS), ethanol (50% w/w EtOH in PBS), and propylene glycol (50% w/w PG in PBS) and found that PBS had the highest Kp value at all sites tested compared to the other vehicles. Jmax provides an estimate for the total penetration of drug per unit time through the skin and was found to be highest when PG was used [35]. However, understanding the factors that influence drug absorption from the canine EAC is a subject area requiring further study.

**Table 8:** The Formulations Systems and Major Excipients of the Selected Commercial COE Products

Product No	Product name	Formulation system	Major ingredients in the formulation	Reference
1	Surolan	Nonaqueous system	silica colloidal anhydrous and liquid paraffin	Summary of product characteristics (Elanco Europe Ltd)
2	Dermotic	Nonaqueous system	paraffin oils and paraffin wax	Summary of product characteristics (Troy Laboratories Pty Ltd)
3	Mometamax	Nonaqueous system	mineral oil polyethylene	Product material safety data (Intervet Inc.)
4	Easotic	Nonaqueous system	liquid paraffin	Summary of product characteristics (Virbac)
5	Canaural	Nonaqueous system	sesame oil	Summary of product characteristics (Dechra)
6	Osurnia	Nonaqueous system	butylhydroxytoluene (e 321) hypromellose lecithin oleic acid propylene carbonate glycerol formal	Summary of product characteristics (Elanco Europe Ltd)
7	Baytril Otic	emulsion system	benzyl alcohol cetylstearyl alcohol a neutral oil purified water emulsion	Product description (Bayer HealthCare LLC)
8	Aurizon Ear Drops	Nonaqueous system	propyl gallate (e310), sorbitan oleate silica, colloidal hydrophobic triglycerides, medium-chain	Summary of product characteristics (Vetoquinol UK Limited)
9	Apex PMP	Nonaqueous system^a^	-	Product material safety data sheet (Apex Laboratories Pty Ltd),

^a^ Assumption made based on the formulation density (0.84 – 0.89 g/mL)

Another interesting factor that can influences drug penetration is the physicochemical properties of the active pharmaceutical ingredient. Drug molecules with molecular weight < 400, log P between -1.0 to 4, and a permeability coefficient of > 0.5 × 10^–3^ cm/h [36] can readily penetrate the skin. Thus, it is possible there may be some systemic exposure when these drugs are administered topically. However, we did not investigate the impact of cerumen commonly found in canine’s EAC and its effect on drug absorption. Preventing drug penetration is especially important for medications with ototoxic effects. Aminoglycosides (for example, amikacin, gentamicin, and apramycin) are well known for their potential for ototoxic effects. Ototoxic effects of aminoglycosides are possible through all routes of administration including systemic routes and topical application to the ear [37] but there may be species differences in susceptibility to gentamicin ototoxicity as observed by Strain et al. in dogs [38].

## Conclusion and future direction

A significant difference was observed in viscosity, syringeability, adhesion, and droplet size of the evaluated commercial otic products. As previously mentioned, viscosity and spreadability are inversely related, therefore it is important that these properties are balanced in order to obtain ideal values for each [14]. The results from the viscosity tests suggest that a viscosity value of less than 0.5 Pa.s with a torque value of 150 – 250 uNm is ideal in order to achieve optimal spreadability [39]. Accordingly, Baytril Otic and Derm Otic showed the ideal viscosity and spreadability values. Products containing polymers exhibited better bioadhesive properties compared to products that didn’t have polymers. The ease of application was assessed by measuring the syringeability and drop size of the COE products. The less force required to expel the product through a syringe indicates greater ease of application and the smaller variation of drop size leads to better dose accuracy. Baytril Otic presented the smallest syringeability force of 137 g which suggests it would be the easiest product to apply with a syringe. Derm Otic, Baytril Otic and Aurizon Ear Drops’s variation of drop size was less than 2. Since large variation of formulation characteristics exists amongst commerically available products, further studies that examine the effect of the difference on clinical effect might be required.

A major consideration during drug development for veterinary purposes is owner compliance which is very much dependent on the ease and frequency of product administration [40]. Owner compliance has proven to be an issue upon application of topical otic products especially because these medications can be difficult to administer [41] particularly when multiple doses are needed. The ease of the application allows for better compliance [41]. Hence, it is important to take ease of application into consideration when developing products to treat COE in order for the target dog to receive the correct dose of the drug for the duration of treatment.

## Supplementary Information

**Additional file 1: Table S1.** The viscosity of selected commercial otic products at different temperatures (mean ± SD, *n*=3). **Table S2.** Physicochemical Properties of Selected Commercial COE products and test statistics.

## Data Availability

All generated data is included in this article. The corresponding author can be contacted if further information is required about the data.
