# Supplementary material for: Physicochemical properties of otic products for Canine Otitis Externa: comparative analysis of marketed products
Source: BMC Vet Res. 2023 Feb 9;19:39. doi: 10.1186/s12917-023-03596-2 (PMC9909939; doi:10.1186/s12917-023-03596-2)
Supplement: Supplementary file 1 — Additional file 1: Table S1. The viscosity of selected commercial otic products at different temperatures (mean ± SD, n=3). Table S2. Physicochemical Properties of Selected Commercial COE products and test statistics. [file 12917_2023_3596_MOESM1_ESM.docx]

**Table S1. The viscosity of selected commercial otic products at two different temperatures (mean ± SD, n=3)**

| Product No. | Product  Name | Temperature (^o^C) | Viscosity  (Pa.s) | Torque  (uNm) | Shear Rate  (1/s) | RPM |
| --- | --- | --- | --- | --- | --- | --- |
| 1 | Surolan | 24.99 ± 0.01 | 2.76 ± 0.20 | 244.13 ± 17.58 | 100.02 ± 0.00 | 84.90 ± 0.00 |
|  |  | 38.89 ± 0.01 | 1.15 ± 0.09 | 203.96 ± 16.06 | 200.04 ± 0.00 | 169.80 ± 0.00 |
| 2 | Derm Otic | 25.00 ± 0.01 | 0.45 ± 0.02 | 238.03 ± 11.83 | 600.01 ± 0.00 | 509.30 ± 0.00 |
|  |  | 38.89 ± 0.01 | 0.18 ± 0.01 | 191.29 ± 7.34 | 1200.01 ± 0.00 | 1018.60 ± 0.00 |
| 3 | Mometamax | 25.00 ± 0.01 | 1.93 ± 0.05 | 426.50 ± 11.62 | 249.99 ± 0.00 | 212.20 ± 0.00 |
|  |  | 38.89 ± 0.01 | 1.01 ± 0.09 | 447.49 ± 40.45 | 499.98 ± 0.00 | 424.40 ± 0.00 |
| 4 | Easotic | 24.99 ± 0.01 | 0.95 ± 0.06 | 250.88 ± 15.29 | 299.94 ± 0.00 | 254.60 ± 0.00 |
|  |  | 38.90 ± 0.01 | 0.37 ± 0.04 | 198.02 ± 22.09 | 600.01 ± 0.00 | 509.30 ± 0.00 |
| 5 | Canaural | 24.99 ± 0.01 | 0.69 ± 0.01 | 304.41 ± 3.06 | 499.98 ± 0.00 | 424.40 ± 0.00 |
|  |  | 38.89 ± 0.01 | 0.48 ± 0.01 | 422.83 ± 5.14 | 999.97 ± 0.00 | 848.80 ± 0.00 |
| 6 | Osurnia | 3.98 ± 0.00 | 15.48 ± 0.96 | 1367.73 ± 84.99 | 100.02 ± 0.00 | 84.90 ± 0.00 |
|  |  | 24.99 ± 0.01 | 4.68 ± 0.09 | 413.88 ± 7.64 | 100.02 ± 0.00 | 84.90 ± 0.00 |
|  |  | 38.90 ± 0.01 | 2.40± 0.11 | 212.48 ± 9.08 | 100.02± 0.00 | 84.90 ± 0.00 |
| 7 | Baytril Otic | 24.99 ± 0.01 | 0.31 ± 0.02 | 190.54 ± 12.81 | 700.03 ± 0.00 | 594.20 ± 0.00 |
|  |  | 38.90 ± 0.02 | 0.15 ± 0.02 | 130.48 ± 14.45 | 999.97 ± 0.00 | 848.80 ± 0.00 |
| 8 | Aurizon Ear Drops | 25.00 ± 0.01 | 0.94 ± 0.13 | 291.34 ± 41.35 | 350.01 ± 0.00 | 297.10 ± 0.00 |
|  |  | 38.90 ± 0.01 | 0.75 ± 0.14 | 462.39 ± 88.74 | 700.03 ± 0.00 | 594.20 ± 0.00 |
| 9 | Apex BMP | 24.99 ± 0.01 | 0.61 ± 0.04 | 271.11 ± 19.26 | 499.98 ± 0.00 | 424.40 ± 0.00 |
|  |  | 38.89 ± 0.02 | 0.39 ± 0.02 | 344.38 ± 19.88 | 999.97 ± 0.00 | 848.80 ± 0.00 |

**Table S2: Physicochemical Properties of Selected Commercial COE products and test statistics.**

| Properties | Surolan | Dermotic | Mometamax | Easotic | Canaural | Osurnia | Baytril Otic | Aurizon Ear Drops | Apex PMP | *P-value* |
| --- | --- | --- | --- | --- | --- | --- | --- | --- | --- | --- |
| Detachment Force (g) | 38.0 ± 7.6 | 69.4 ± 8.1 | 77.9 ± 7.9 | 33.1 ± 3.0 | 29.7 ± 7.2 | 124.3 ± 25.5 | 14.6 ± 2.4 | 17.4 ± 5.1 | 37.2 ± 2.7 | <0.001 |
| Spreadability (mm) | 54 ± 0.6 | 66 ± 2.5 | 56 ± 4.5 | 46 ± 1.5 | 79 ± 1.5 | 35 ± 1.2 | 69 ± 7.2 | 50 ± 2.6 | 52 ± 1.0 | 0.452 |
| Syringeability Force (g) | 293 ± 43 | 1546 ± 155 | 970 ± 469 | 1400 ± 254 | 377 ± 165 | 141 ± 22 | 137 ± 7 | 1199 ± 51 | 1028 ± 248 | <0.001 |
| Droplet Size (mg/drop) | 23 ± 3.8 | 34 ± 1.7 | 20 ± 3.4 | 27 ± 3.9 | 25 ± 3.7 | 30 ± 6.2 | 28 ± 1.4 | 28 ± 1.6 | 26 ± 4.7 | <0.001 |

*The statistics were performed using one-way anova
